# Photocatalytic Degradation of Organic Dyes and Antimicrobial Activities by Polyaniline–Nitrogen-Doped Carbon Dot Nanocomposite

**DOI:** 10.3390/nano11051128

**Published:** 2021-04-27

**Authors:** Moorthy Maruthapandi, Arumugam Saravanan, Priyanka Manohar, John H. T. Luong, Aharon Gedanken

**Affiliations:** 1 Department of Chemistry, Bar-Ilan Institute for Nanotechnology and Advanced Materials, Bar-Ilan University, Ramat-Gan 52900, Israel; lewismartin.jesus@gmail.com (M.M.); saran.bc94@gmail.com (A.S.); 2 Department of Chemistry, School of Chemical and Biotechnology, Sastra University, Thanjavur 612001, India; priyankasumathi6@gmail.com; 3 School of Chemistry, University College Cork, T12 YN60 Cork, Ireland; luongprof@gmail.com

**Keywords:** PANI-N@CDs, nanocomposite, sonochemical method, dye degradation, antimicrobial activity

## Abstract

Nitrogen-doped carbon nanodots (N@CDs) were prepared by hydrothermal processing of bovine serum albumin (Mw: 69,324 with 607 amino acids). A polyaniline (PANI-N@CDs) nanocomposite was then synthesized by ultrasonication and used to degrade Congo red (CR), methylene blue (MB), Rhodamine B (RhB), and crystal violet (CV) four common organic dyes. The PANI-N@CD nanocomposite simultaneously adsorbed and concentrated the dye from the bulk solution and degraded the adsorbed dye, resulting in a high rate of dye degradation. The combination of holes (h+), hydroxyl (OH^•^), and O_2_^•−^ was involved in the N@CD-mediated photocatalytic degradation of the dyes. Under visible light illumination at neutral pH, the PANI-N@CDs were proven as an efficient adsorbent and photocatalyst for the complete degradation of CR within 20 min. MB and RhB were also degraded but required longer treatment times. These findings supported the design of remediation processes for such dyes and predicted their fate in the environment. The nanocomposite also exhibited antimicrobial activities against Gram-negative bacterium *E. coli* and Gram-positive bacterium *S. aureus*.

## 1. Introduction

Organic dyes are mostly used for coloring cotton, silk, and fibers, and their discharge in wastewater is a critical issue in environmental remediation due to their universal use in many industries. Numerous dyes among 6000 synthetic organic dyes used in the textile industry are harmful and noxious [1,2,3,4]. The textile industries release an enormous amount of wastewater contaminated with toxic chemicals and heavy metals. Thus, the discharge of such polluted effluents becomes a serious environmental issue concerning their long-term and acute effects on the ecosystem [5,6,7].

Conductive polymers and their composites have received great attention for the development of nanocomposites due to their mechanical and optical properties. To address this issue, one of the goals of the polymer nanocomposite preparation has been to degrade the organic dye from wastewater via visible light irradiation. In this aspect, considerable efforts have focused on the synthesis of numerous polymer nanocomposites [8,9,10,11,12], key properties, and applications [13,14,15]. However, applications of conductive polymers have been limited due to their poor mechanical properties. Various metal oxides and other nanomaterials have been synthesized with conductive polymers to prepare composite materials or complexes with enhanced mechanical properties and desirable physical and structural properties [16,17]. Doping conductive polymers with CDs results in considerably enhanced photocatalytic activity of the polymer nanocomposites [18]. CDs have several attractive properties, such as photoluminescence, high mechanical strength, narrow band-gap, and optical transparency, in addition to being biodegradable, biocompatible, and nontoxic [19,20]. Therefore, they have great potential in physical and biomedical applications. Polymers decorated with CDs exhibit enhanced hydrodynamic radii, high density, and high surface area [18]. Besides, the polymer composites of PANI@TiO_2_, PANI/ZnO, PANI/SiO_2_, PANI/ZrO_2_, and PANI/Ag have been used as adsorbents for organic dyes [21,22,23,24,25,26,27]. Although the removal of toxic organic dyes from wastewater by PANI is the first important step [28,29,30,31], a remediation step for such dyes is needed in the treatment of water and wastewater. PANI is commonly used as an adsorbent for anionic dyes and forms a polymer composite as polyaniline-implanted cellulose for effective adsorption of methyl orange and eosin yellow with SO_3_^‒^ and COO^‒^ groups [32]. The polyaniline nanocomposite has invigorated great interest in antibacterial applications due to its biocompatibility, low cost, and simple synthesis method. The mechanical strength and free radical properties were strengthened by incorporating the carbon dots on the polymer. The inclusion of CDs has improved the physical and chemical activity of the polymer. The release of ROS (reactive oxygen species) causes effective oxidative damage to cellular components and leads to cell lysis [33,34,35,36].

This work focuses on the synthesis of a nanocomposite from PANI and N@CDs by simple ultrasonication for the adsorption and degradation of organic molecules. C-dots can act as an electron acceptor or electron donor, a prerequisite for making them a potential catalyst. The novelty of this work is found in the direct deposition of N@CDs on the surface of PANI by ultrasonication, and this strong deposition causes the effective interaction between polymer and N@CDs with a higher surface area of the polymer nanocomposite. The estimated surface areas of PANI and PANI-N@CDs from BET analysis are 1.6 m²/g and 6.000 m²/g, respectively. The degradation of Congo red (CR), methylene blue (MB), and Rhodamine B (RhB) by the PANI-N@CDs under visible light illumination is investigated as the generated hydroxyl radicals would be expected to play an important role in their degradation. A degradation pathway of Congo red is also postulated and supported by pertinent experimental data. The PANI-N@CD composite is also tested as an antimicrobial agent against *E. coli* and *S. aureus.*

## 2. Experimental Section

### 2.1. Preparation of N@CDs and PANI

A hydrothermal Teflon cell containing 0.2 g BSA dissolved in 25 mL of distilled water was kept in a hot oven at 180 °C for 12 h. The product was then filtered and purified by dialysis [37]. The PANI was synthesized as described by Moorthy et al. [37].

### 2.2. Preparation of PANI-N@CDs and Analytics

A reaction solution consisting of 0.2 g of PANI powder, 80 mL of DD water, and 5 mg of N@CDs was kept under sonication for 5–7 min with a 35% amplitude, followed by centrifugation at 2000 rpm for 20 min. The FTIR spectrum was acquired by the Transon 27 spectrometer (Bruker, Karlsruhe, Germany) from 4000 to 400 cm^−1^. The composite and N@CDs morphologies were analyzed using HRSEM (high-resolution scanning electron microscopy) equipped with an FEI Megallon 400 L microscope. For HRSEM measurement, detailed information on the procedure and instrumentation is available elsewhere [38].

### 2.3. Degradation of Dye under Visible Light Irradiation

The degradation of CR or other dyes by PANI-N@CDs under visible light illumination was carried out at pH 6.3 and room temperature. A solution consisting of 10 mg of the dyes and 50 mg of PANI-N@CDs in 100 mL of deionized water was prepared in the dark and then subjected to visible light. The sample (2 mL) collected during degradation was syringe-filtered to provide a clear solution for the absorbance measurement.

### 2.4. Seawater Experiment

Seawater was collected from Tel Aviv beach, Israel, and used as a “real-world” sample. On average, seawater has a salinity of about 3.5% (35 g/L or 599 mM of NaCl). In brief, 10 mg dye was dissolved in 100 mL seawater; to this, 50 mg of PANI-N@CDs was added. The whole preparation was conducted in the dark, and the degradation of the dyes was performed under visible light. The testing sample (3 mL) was collected at various contact times during the degradation process to measure the resulting UV absorbance of the dyes.

## 3. Result and Discussion

**Figure 1:**
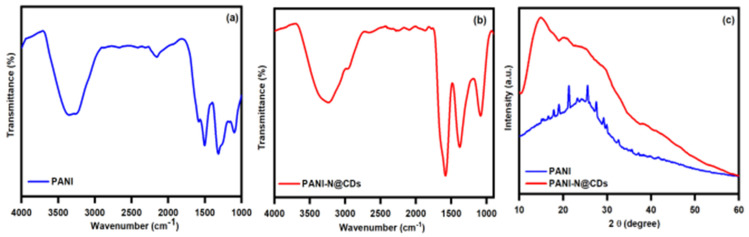
(**a**,**b**) FTIR spectra of PANI and PANI-N@CDs; (**c**) XRD pattern of PANI and PANI-N@CDs.

The FTIR spectra of PANI and PANI-N@CDs are shown in Figure 1. The spectra depict two broad bands at 3304 and 3321 cm^−1^, which are assigned as the –N–H stretching vibration [39]. The O–H stretching vibration is ascribed to the peak at 3364 cm^−1^, whereas two counterparts at 1317 and 1376 cm^−1^ are attributed to the C=N stretching vibration [40]. The bands at 1583 and 1586 cm^−1^ are denoted as the –N–H asymmetrical stretching vibration and the C=C stretching vibration is evinced by the 1506 cm^−1^ peak [39]. The bands at 2156 and 2953 cm^−1^ indicate the aromatic C=C stretching vibration and C–H aliphatic and aromatic stretching vibration [41]. The peaks at 1088 and 1100 cm^−1^ are due to the C–O stretching vibration and =C–H in-plane vibration [42]. The PANI-N@CDs show strong broad bands compared to PANI due to the incorporation of N@CDs. The N–H stretching vibration and C–N stretching vibration peaks were also shifted noticeably to higher wavenumbers. The absorption frequencies and their corresponding functional groups are displayed in Table 1.

The XRD diffraction patterns of PANI and PANI-N@CDs are displayed in Figure 1c. PANI shows broad diffraction peaks between 15 and 30°, reflecting the parallel and perpendicular periodicity of the PANI chain. PANI exhibits a few crystalline peaks due to the replication of the quinoid and benzenoid rings of the polymer. The XRD of the PANI-N@CDs demonstrated that the crystalline peaks disappeared owing to the formation of the N@CDs and the polymer nanocomposite is amorphous, whereas the PANI is semi-crystalline. The N@CDs can be grafted on the polymer surface due to the strong ultrasonic method, a rationale behind the disappearance of the crystalline peak in the polymer nanocomposites. A sharp broad peak at ~16° was observed, indicating that N@CDs formed a strong interaction with the polymer backbone in the polymer chain.

**Figure 2:**
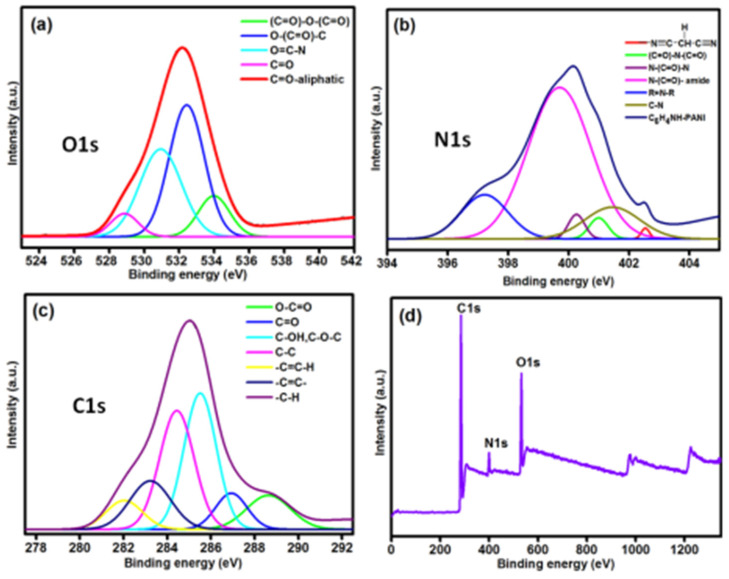
High-resolution O1s, N1s, and C1s (**a**–**c**) spectra; and (**d**) full XPS survey of PANI-N@CDs.

The XPS spectra of PANI-N@CD nanocomposites indicate the various elements in the polymer and its composites. XPS spectra of PANI-N@CDs indicated the presence of C, N, and O (Figure 2). The high-resolution XPS spectra of PANI-N@CDs confirmed the presence of various functional groups in the polymer nanocomposites. The high-resolution spectra and XPS survey for PANI are provided in the Supporting Information (Figure S1).

### 3.1. Morphology Studies

**Figure 3:**
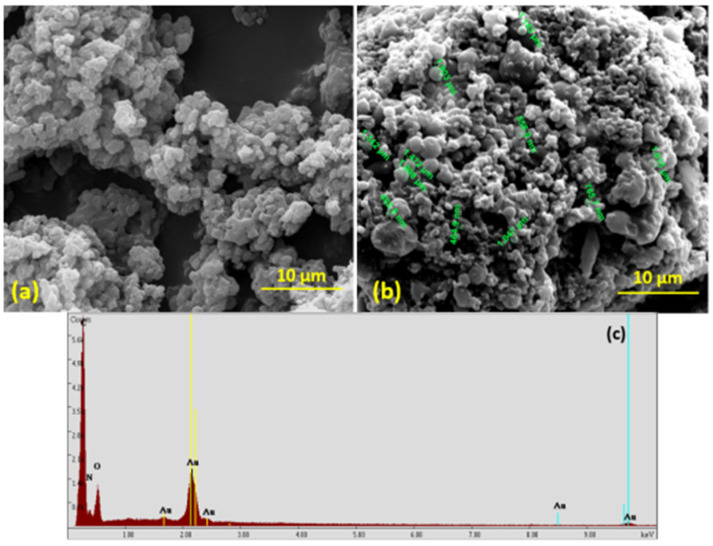
SEM images of (**a**) PANI and (**b**) PANI-N@CDs; (**c**) SEM-EDX of PANI-N@CDs.

PANI exhibited agglomerated irregular shapes with an average size of ~10 µm and broad size distribution (Figure 3a). The PANI-N@CDs also displayed agglomerated irregular shapes with 10 µm size together with agglomerated N@CD spherical-shaped particles of 200–750 nm (Figure 3b). No discrete spherical particles were observed on the polymer nanocomposite, illustrating that the N@CDs strongly interacted with the polymer backbone by ultrasonication. Note that SEM imaging of N@CDs is available in the literature [43]. The EDX spectrum and SEM-EDX elemental mapping spectrum of the polymer nanocomposites display the elementary components C, N, O, and Au with an even distribution (Figure 3c).

**Figure 4:**
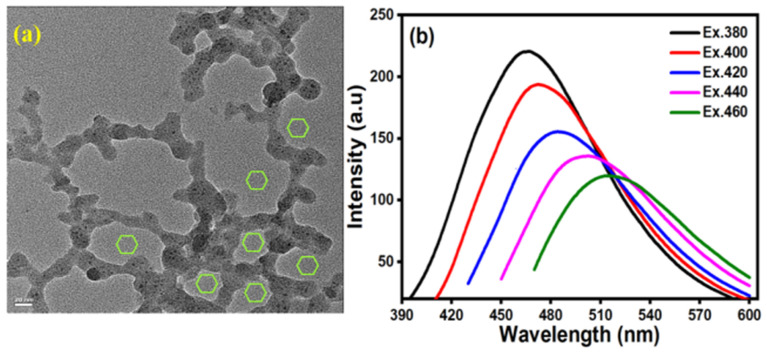
(**a**) TEM image of PANI-N@CDs; (**b**) fluorescence spectra of PANI-N@CDs.

The particle size of the polymer nanocomposite estimated by TEM imaging ranged from 20 to 70 nm, due to the agglomeration of the PANI rings with N@CDs. A PANI aromatic ring structure together with N@CD spherical-shaped particles resulted in the formation of a stable complex between N@CDs and PANI (Figure 4a). The fluorescence emission spectra were analyzed for the polymer nanocomposite to observe if there were any spectral changes after the formation of PANI-N@CDs (Figure 4b). There were no changes in the peak position; however, the intensity was gradually reduced by a factor of 4 due to the formation of PANI-N@CDs [38]. Nevertheless, N@CDs still retained noticeable fluorescence after their formation of a nanocomposite with PANI.

### 3.2. Degradation Kinetics

A series of experiments was conducted to assess the capability of PANI-N@CDs to degrade four common organic dyes. No degradation was observed after 20 min when the experiments were conducted in the dark (data not shown). Under visible light irradiation, CR was completely degraded after 20 min, and 60% and 20% degradation were observed for MB and RhB, respectively, whereas only 2–3% of CV was degraded. For comparison, the photodegradation of CR by ZnO at 365 nm was achieved only after 60 min and the efficiency was 95% [44]. Silver nanoparticles can effectively degrade methylene blue by 95% but require 72 h of exposure time [45]. For effective degradation of other dyes, e.g., crystal violet (CV), CDs must be conjugated with TiO_2_ to improve the photocatalytic activity as the degradation of this dye at 20 ppm is over 97% under UV illumination for 105 min with anatase [46]. In this study, CV exhibited strong resistance to degradation. Of note is the development of visible-light-active nanosized Ag_2_S–ZnS loaded on cellulose for the degradation of RhB [47]. Under simulated sunlight, 30 ppm of this dye is oxidized using 30 mg of the composite dose in the pH range 4–12 within 90 min. However, 4 mM of H_2_O_2_ is required for the maximum degradation of the dye. In our study, a neutral pH was used to carry out the degradation to avoid the use of acid or base. The degradation of CR by ZnO is more effective at pH 8–9, compared with the performance at neutral pH [44].

**Figure 5:**
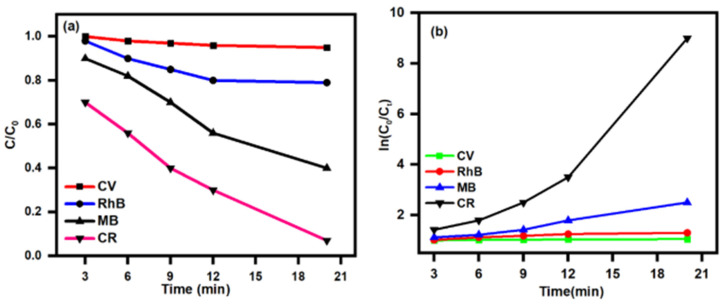
(**a**) Photocatalytic degradation of four common dyes under visible light; (**b**) pseudo-first-order model.

The degradation kinetics is governed by the pseudo-first-order (PFO) equation ln *C*_0_/*C_t_* = *kt*, where *C*_0_ is the initial dye concentration, *C_t_* is the dye concentration at a specific time (*t*), and *k* is the rate constant of the PFO reaction. The rate of degradation of organic dyes onto PANI-N@CDs nanocomposite under the irritation of visible light is displayed in Figure 5a. The PFO reaction is shown in Figure 5b. The photocatalytic degradation reaction of dye under visible and dark light is provided in the SI (Figure S2). The degradation efficiency is calculated as *R*_deg_ = 100(*C*_0_ − *C_f_*)/*C*_0_, where *C_f_* (mg/L) is the final concentration of the organic dye in the solution. The *R*_deg_ value was estimated to be 97% for CR, 61 for MB, 38 for RhB, and only 16 for CV. The rate of degradation is provided in Figure S3 (SI). The photocatalytic degradation result for seawater is provided in the Supporting Information (SI). The degradation was followed by the UV absorbance at different contact times, and the complete degradation is shown in Figure S4.

The fluorescence of N@CDs can effectively be quenched by electron-accepting or electron-donating quencher, unraveling their role as electron donors and acceptors. CDs under visible light play an important role in the degradation of the four organic dyes tested in this study. CDs can generate hydroxy radical (OH^•^) under visible light, as proven by an experiment conducted with TiO_2_ and TiO_2_ decorated with nitrogen-doped carbon dots [48]. N@CDs expand the photocatalytic response of anatase beyond the UV region and co-generate hydroxyl radicals in the visible–NIR range [48]. The TiO_2_-N@CDs under white LED, a combination of 450 and 550 nm, also exhibit the highest photoactivity, compared with the other LEDs, e.g., red (740) or green (532 nm) [48,49]. This deployment might be necessary for the degradation of more recalcitrant dyes and organic pollutants to exploit the synergism of the anatase CDs. The photocatalytic ability of semiconductor is significantly dependent on their band gap values with the following order: ZnO > TiO_2_ > CeO_2_ > Al_2_O_3_ > Fe_2_O_3_ [45]_._ The production of ROS (OH^−^, H_2_O_2_, and O_2_^2−^) on the ZnO surface is feasible as the valence band electrons jump to the conduction band when ZnO is under incident radiation with photons of energy above 3.3 eV [46,47]. Consequently, positive holes (h^+^) are formed in the VB to serve as a direct oxidant for generating reactive hydroxyl radicals (OH^•^) [50,51,52]. The ^•^O_2_ generation by CDs under 800 nm laser ablation has also been reported with a quantum yield of 7.2% [53]. Therefore, there was a combined involvement of holes (h^+^), hydroxyl (OH^•^), and O_2_^•−^ in the N@CD-mediated photocatalytic degradation of the dyes. The PANI-N@CD nanocomposite simultaneously adsorbed the dye from the bulk solution and degraded the adsorbed dye, resulting in the high rate of degradation of the dye. The stepwise photocatalytic mechanism for the generation of ROS is complicated [44]; however, it can be simplified as follows:

N@CDs + hν → N@CDs [e^−^_(CB)_ + h^+^_(VB)_] (1)

H_2_O + h^+^ → ^•^OH_ads_ + H^+^ (2)

Dyes + ^•^OH_ads_ → Oxidized dyes (3)

The photogeneration of hydroxyl radicals (^•^OH), critical oxidants, induces the redox decomposition of various organic contaminants and plays a key role in biological processes [54,55,56,57].

**Scheme 1:**
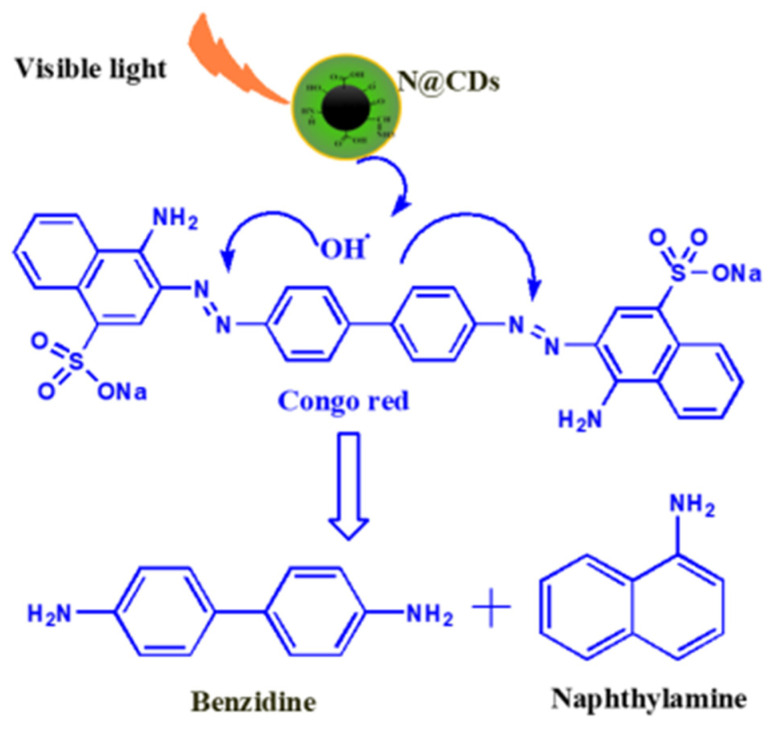
Congo red is degraded to benzine and naphthylamine when the dye is subject to visible light in the presence of PANI-N@CDs. The OH^.^ radical generated by N@CDs can break down this dye to benzine and naphthylamine.

**Figure 6:**
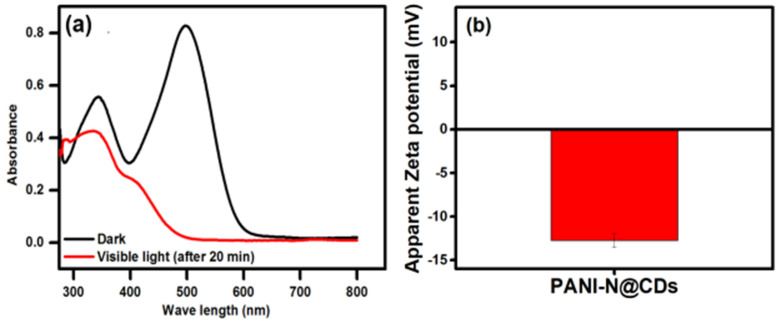
(**a**) Absorbance of Congo red in the presence of PANI-N@CDs in the dark and under visible light illumination; (**b**) zeta potential of PANI-N@CDs (average ± SD of five measurements).

Rapid degradation of CR was an important finding because this benzidinediazo-bis-1-naphthylamine-4-sulfonic acid dye exhibits carcinogenic properties [58]. Some fungi are capable of degrading CR to benzidine and naphthylamine [59]. A plausible transformation of CR into benzidine and naphthylamine was investigated by probing the absorbance of CR after 20 min of treatment with PANI-N@CDs under visible light illumination (Scheme 1). The sharp absorbance peak at 498 nm almost disappeared, and the second peak at ~355 nm was also reduced appreciably (Figure 6). A peak that emerged at 280 nm could be assigned to the formation of benzidine, whereas naphthylamine with a broad absorption peak ranging from 310 to 330 nm was overlapped with the diminishing CR peak at 355 nm. This overlap was the reason why the CR peak at 355 nm did not completely disappear, as shown in Figure 6.

### 3.3. Antibacterial Activity Test

Gram-positive *S. aureus* and Gram-negative *E. coli* were grown using lysogeny broth at 37 °C overnight with agitation at 150 rpm. The bacteria were collected for the absorbance (at 595 nm (OD595)) measurement during incubation. The resulting bacterial solution was adjusted to 10^7^; from this, 500 μL of the bacterial solution was mixed with 500 μL of each sample (PANI, N@CDs, and PANI-N@CDs) and then incubated for 24 h in a shaker at 150 rpm and 37 °C. Then, 100 μL of each sample was serially diluted in 96-well plates, and 10 μL of the solution was plated on an agar plate. The colony-forming unit (CFU) method was used to calculate the rate of bacterial growth.

### 3.4. Antibacterial Activity

**Figure 7:**
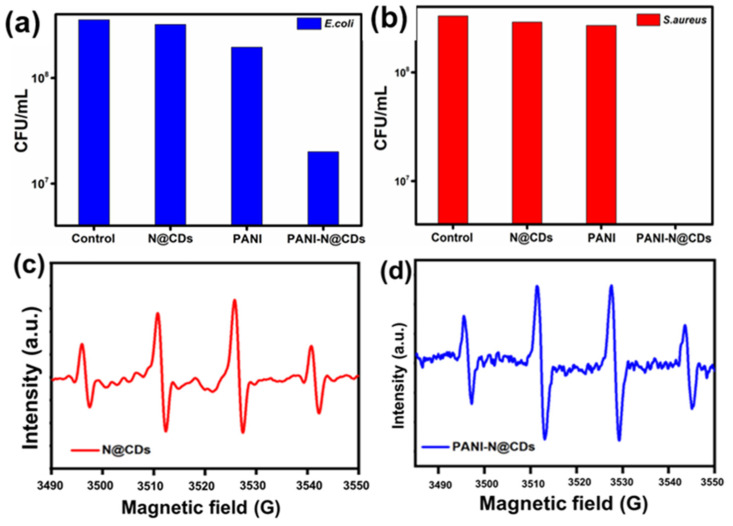
Antibacterial treatment of N@CDs, PANI, and PANI-N@CDs against (**a**) *E. coli* and (**b**) *S. aureus*; (**c**,**d**) EPR spectra for N@CDs and PANI-N@CDs.

The PANI-N@CD nanocomposite was investigated for its antimicrobial activity against the two common pathogens *E. coli* and *S. aureus*, whereas PANI and CDs served as two controls for comparison. The minimum inhibitory concentration (MIC) of the nanocomposite was determined to be 1000 µg/mL for *E. coli* and 750 µg/mL for *S. aureus*. The superior activity of PANI-N@CDs on growth inhibition was found at 1000 µg/mL within 24 h for *S. aureus* and *E. coli* (Figure 7). The mechanism for cell death could be related to the release of reactive oxygen species (ROS) and the role of the surface charge of the nanocomposite. The ROS generation was analyzed by electron paramagnetic resonance for the materials. The EPR spectra of N@CDs and PANI-N@CDs are provided in Figure 7c,d. The DMPO was used as a spin trap to detect OH and superoxide radicals. ROS generation by PANI and N@CDs played a major role in the eradication of bacteria. The unpaired or free electrons in PANI-N@CDs can react with the dissolved oxygen in the solution to produce additional ROS. The free radical properties of PANI are shown in our previous publications [33,34,35,36]. The synthesized PANI-N@CDs have a negative charge on their surface due to the presence of various functional groups, as confirmed by XPS spectra (Figure 2). The PANI-N@CDs have a zeta potential of −13.6 ± 0.8 mV (Figure 6b); consequently, electrostatic interactions play an important role in the eradication of the tested bacteria. The Gram-positive bacteria have membranes with positive charges, However, there is a strong interaction between negatively charged surfaces of PANI-N@CDS and Gram-positive bacteria. Various forces could also be involved in the interaction such as van der Waals forces, London dispersion forces, and stronger dipole–dipole forces.

## 4. Conclusions

This work presents a simple method for the synthesis of a PANI-N@CD nanocomposite for the simultaneous adsorption and degradation of four toxic dyes. The dyes are adsorbed on the polymer backbone due to the presence of interacting sites in the polymer nanocomposite. The kinetic data reveal that the PANI-N@CDs nanocomposite is capable of degrading organic molecules under visible light illumination. TiO_2_ or ZnO can be conjugated with N@CDs to form more effective photocatalysts for compounds such as CV and RhB, found to be recalcitrant in this study. In this aspect, LEDs with different wavelengths can be set up toward the development of multiple photochemical-related degradation processes to remediate a wider spectrum of organic dyes and pollutants. The PANI-N@CDs also displayed effective antibacterial activities against *E. coli* and *S. aureus* within 24 h. The release of ROS effectively damaged cell walls and components, whereas the nanocomposite formed a complex with the bacteria and interfered with the diffusion of nutrients and wastes in and out of the cytosol.

## Figures and Tables

**Table 1:** The major functional groups and their corresponding absorption frequency.

Material	Absorption Region (cm^−1^) and Functional Groups	Ref.
PANI	−3005, N–H stretching vibration −2156, C=C aromatic stretching vibration −1586, N-H asymmetrical stretching vibration −1317, C–N stretching vibration −1110, = C-H in-plane vibration	[39,40,41,42]
PANI-N@CDs	−3364, O–H stretching vibration −3231, N–H stretching vibration −2954, C–H aliphatic and aromatic stretching vibration −1583, N–H asymmetrical stretching vibration −1376, C–N stretching vibration −1088, C–O symmetric stretching vibration	

## Data Availability

Data presented in this study are available by requesting from the corresponding author.

## Supplementary Materials

The following are available online at https://www.mdpi.com/article/10.3390/nano11051128/s1. Figure S1. XPS spectra of PANI. Figure S2. Photocatalytic degradation of CR under visible light and dark conditions. Figure S3. Photocatalytic degradation percentage of CR in various periods and dyes. Figure S4. Photocatalytic degradation of CR in real samples.
