# Supplementary material for: Photocatalytic Degradation of Organic Dyes and Antimicrobial Activities by Polyaniline–Nitrogen-Doped Carbon Dot Nanocomposite
Source: Nanomaterials (Basel). 2021 Apr 27;11(5):1128. doi: 10.3390/nano11051128 (PMC8145885; doi:10.3390/nano11051128)
Supplement: Supplementary file 1 [file nanomaterials-11-01128-s001.zip › nanomaterials-1191882 SI updated.pdf]

nanomaterials

Support information

Photocatalytic Degradation of Organic Dyes and Antimicrobial Activities by Polyaniline–Nitrogen-Doped Carbon Dot Nanocomposite

Moorthy Maruthapandi [1], Arumugam Saravanan [1], Priyanka Manohar [2], John H.T. Luong [3] and Aharon Gedanken [1,*]

[1] Department of Chemistry, Bar-Ilan Institute for Nanotechnology and Advanced Materials, Bar-Ilan University, Ramat-Gan 52900, Israel; lewismartin.jesus@gmail.com (M.M.); saran.bc94@gmail.com (A.S.)
[2] Department of Chemistry, School of Chemical and Biotechnology, Sastra University, Thanjavur 612001, India; priyankasumathi6@gmail.com
[3] School of Chemistry, University College Cork, Cork T12 YN60, Ireland; luongprof@gmail.com
* Corresponding author: gedanken@mail.biu.ac.il; Tel: +972-3-531831; Fax: +972-3-7384053

XPS spectra

The XPS spectra of PANI unravel various elements in the polymer. The XPS spectra of PANI show the presence of C, N, and O (Figure S1). The high resolution XPS spectra for N, O and C provides the information about the various functional groups in the polymer.

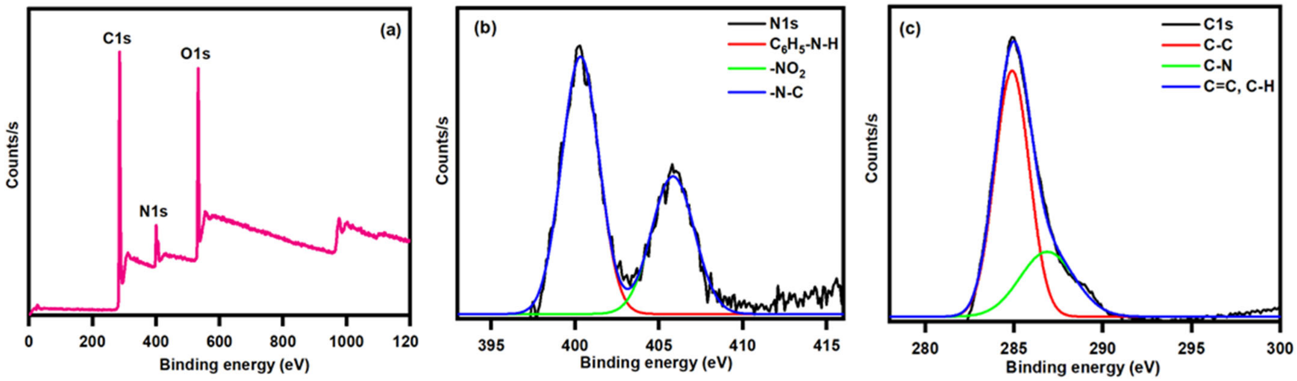

Figure S1. (a) XPS spectra of PANI and (b,c) high-resolution N1s and C1s spectra of PANI.

Kinetic studies

The Pseudo first order of photocatalytic degradation of CR is presented in Figure S2. The kinetic results demonstrated that the degradation obeys the pseudo first-order reaction, which is explained by the following equation (1);

$$\ln \frac{C^0}{C_t} = kt \quad (1)$$

Copyright: © 2021 by the authors. Licensee MDPI, Basel, Switzerland. This article is an open access article distributed under the terms and conditions of the Creative Commons Attribution (CC BY) license (http://creativecommons.org/licenses/by/4.0/).

where C_0 and C_t are the initial and final concentration, time t min, *k* is the rate constant of the pseudo first-order reaction.

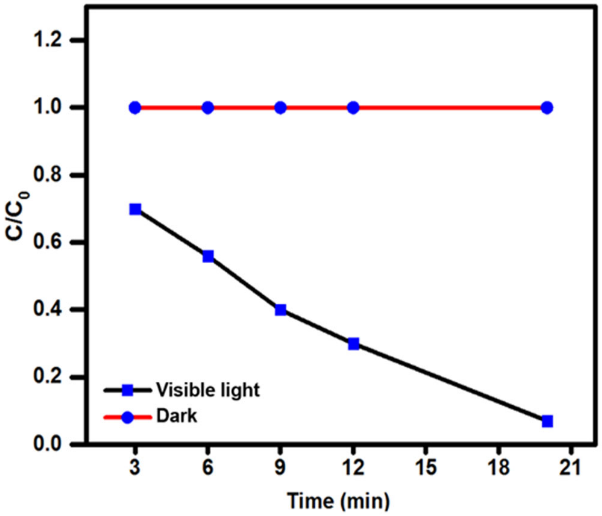

Figure S2. Photocatalytic degradation of CR under visible light and dark condition.

After several contact times with the catalyst (3–15 min), 2 mL of the resulting suspension containing dye was reserved by a filter syringe to provide a clear solution for UV visible absorbance measurement. Dehydration efficiency is calculated by equation (2).

$$R_{deg} = 100(C_{\circ} - C_{f})/C_{\circ} \quad (2)$$

Where the volume of the dye and C_o and C_f (mg/L) are the initial and final concentrations of the organic dye, respectively. *C*(*t*) is the dye concentration in the solution at any time *t* (Figure S3b).

The photocatalytic activity was carried out for various organic dyes such as crystal violet (CV), rhodamine B (RhB), and methylene blue (MB), there is no much degradation was observed on CV, RhB, and MB by PANI-N@CDs under visible light illumination. The degradation result reveals that the polymer nanocomposite selectively degrading CR under visible light irradiation with maximum degradation efficiency. The degradation (%) for various dyes was provided in FigureS3b.

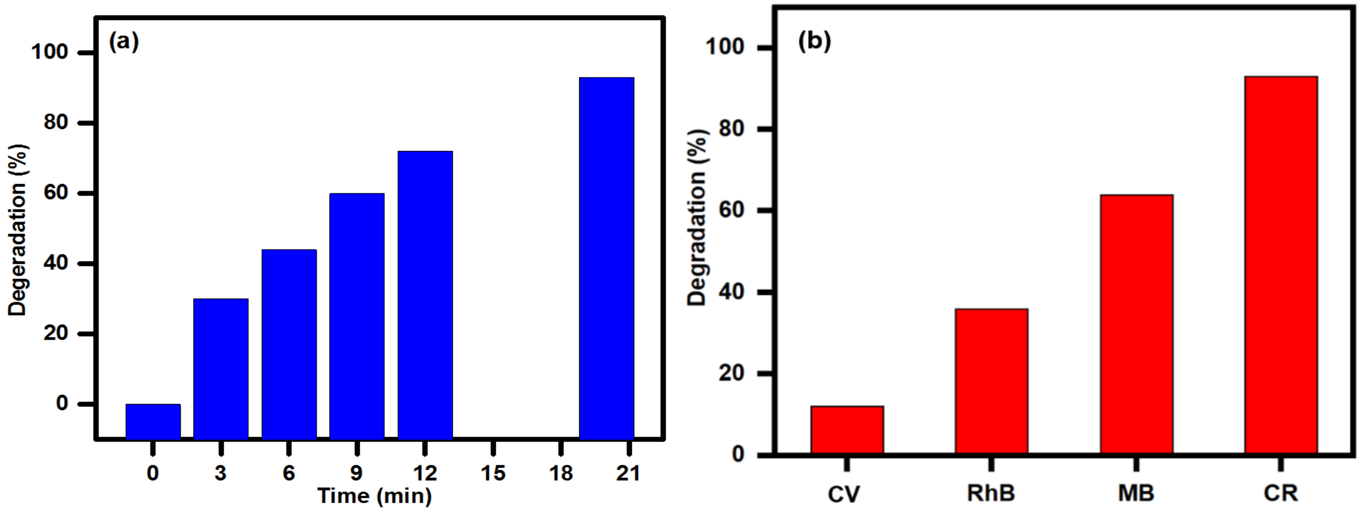

Figure S3. (**a**) Photocatalytic degradation percentage of CR in various periods and (**b**) photocatalytic degradation of various dyes.

Photocatalytic degradation in sea water

After various contact time (3–20 min) the degradation sample was collected to measure the degradation concentration. 2 mL of the resulting suspension containing dye was reserved by a filter syringe to provide a clear solution for UV visible absorbance measurement. Figure S4 shows the complete degradation of CR in sea water after 20 min. Thus, our synthesized polymer nanocomposite can be used as adsorbent for waste water (sea water). The UV-result indicates the complete degradation with formation of other complex with adsorbent.

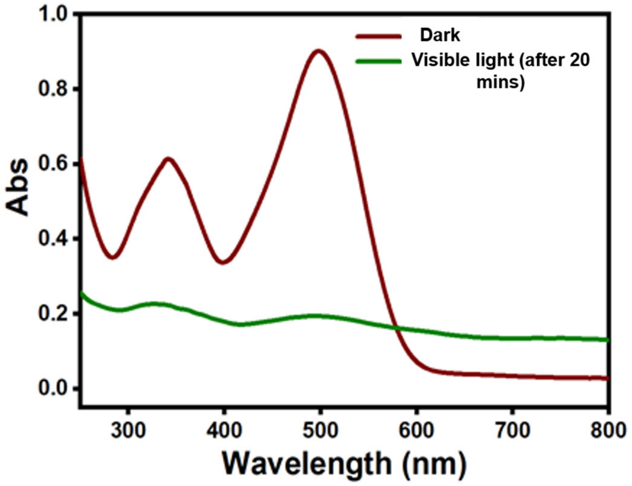

Figure S4. The absorbance of Congo red in sea water the presence of PANI-N@CDs in the dark and under visible light illumination.
